# Supplementary material for: Photoactivation of Color Centers Induced by CW Laser Irradiation in Ion-Implanted Diamond
Source: ACS Photonics. 2025 Jul 1;12(7):3803–14. doi: 10.1021/acsphotonics.5c00826 (PMC12272690; doi:10.1021/acsphotonics.5c00826)
Supplement: Supplementary file 1 [file ph5c00826_si_001.pdf]

Supporting Information

Photoactivation of color centers induced by CW laser irradiation in ion-implanted diamond

Vanna Pugliese[1], Elena Nieto Hernández[1,#], Emilio Corte[1], Marco Govoni[2], Sviatoslav Ditalia Tchernij[1], Paolo Olivero[1], Jacopo Forneris[1]

[1] *Physics Department, University of Torino, 10125 Torino Italy, and Istituto Nazionale di Fisica Nucleare (INFN), Sezione di Torino, 10125 Torino, Italy*

[2] *Department of Physics, Computer Science and Mathematics, University of Modena and Reggio Emilia, 41125 Modena Italy*

[#] corresponding author: elena.nietohernandez@unito.it

S1 - PL count rate stability

We experimentally confirmed that operating in the "laser readout" mode does not induce any changes when one of the irradiated samples is illuminated under these conditions (i.e., 100 µW power for 2 minutes). As illustrated in **Fig. S1**, the photoluminescence (PL) count trace shows no significant variation, confirming the absence of further center activation upon the employed parameters.

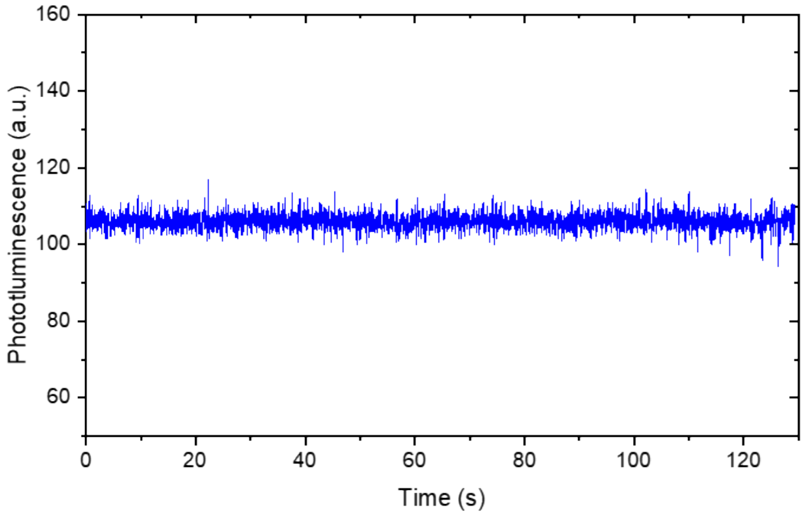

Fig. S1 PL count rate in the MgH^- as a function of time, for a total of 2 min, under 100 µW, 520 nm laser.

S2 - Photoactivation in surface-functionalized diamond

The absence of a Raman shift between the laser engineered spots and the pristine region is evident in **Fig. S2**. Although a slight shift of 0.2 nm appears in the right-hand graph, it falls within the spectral resolution of the experimental setup and therefore lacks statistical significance.

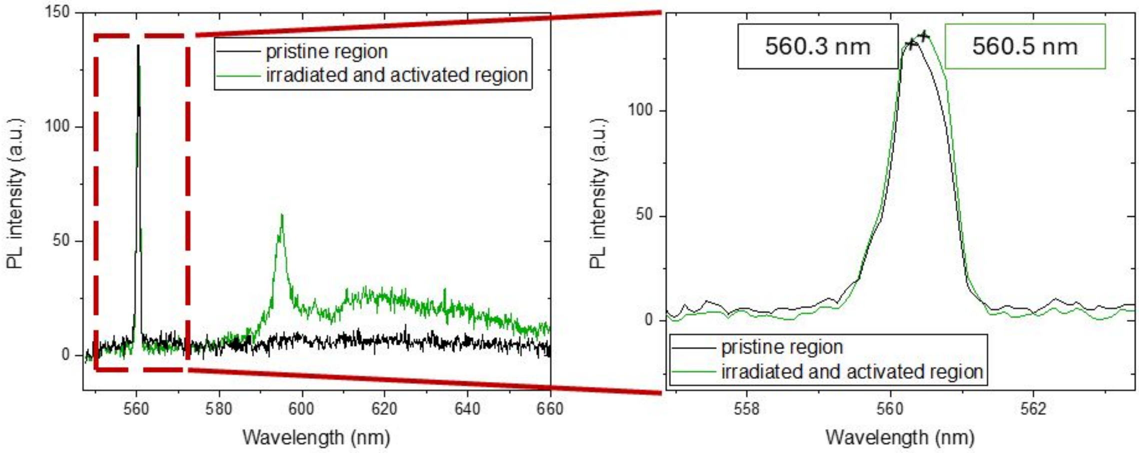

Fig. S2 Spectral comparison between an ion irradiated and laser activated spot in the Sn region and a solely implanted one.

S3 - Photoactivation in surface-functionalized diamond

In order to assess the role of surface chemical functionalization by laser-assisted surface charge modification, two separate regions of a control sample were implanted with MgH^- and Sn^- ions, respectively. The same implantation parameters adopted in the main text were set. Both regions were tested with the same laser processing photoactivation discussed in the present work. In this section we report the data relevant to the 405 nm processing laser, as it was demonstrated (**Figure 5** in the main text) to yield the most efficient activation among the considered wavelengths. Laser processing was performed right after ion implantation without further treatments of the sample using a fixed optical power of 11 mW and 4 different exposure times, namely 1, 5, 15 and 30 minutes. The result is shown in the PL confocal map in **Fig. S3a**. The map (acquired in the region implanted with MgH^- ions using a long pass filter at 550 nm) exhibits an array of bright, photoactivated spots, thus proving the repeatability of the adopted methodology. Subsequently, the surface of the sample was functionalized by means of oxygen plasma treatment (60 Pa pressure, 0.5 sccm O_2 flux, 30 minutes duration, 23 W microwave power) and further investigated by confocal PL microscopy (**Fig. S3b**). No significant differences in the PL features acquired from the same processed region were apparent, indicating that the O-terminated surface did not result in the further activation of MgV^- centers. A further, separate array was finally fabricated by laser processing (same parameters as above) following the plasma treatment. The results of the photoactivation process are shown in **Fig. S3c**, showing that the photoactivation takes place irrespectively of the surface termination of the diamond plate. These data demonstrate that the activation of MgV^- centers cannot be regarded as the effect of laser-assisted surface chemistry modification. Similar results were obtained in the Sn^- implanted region of the sample.

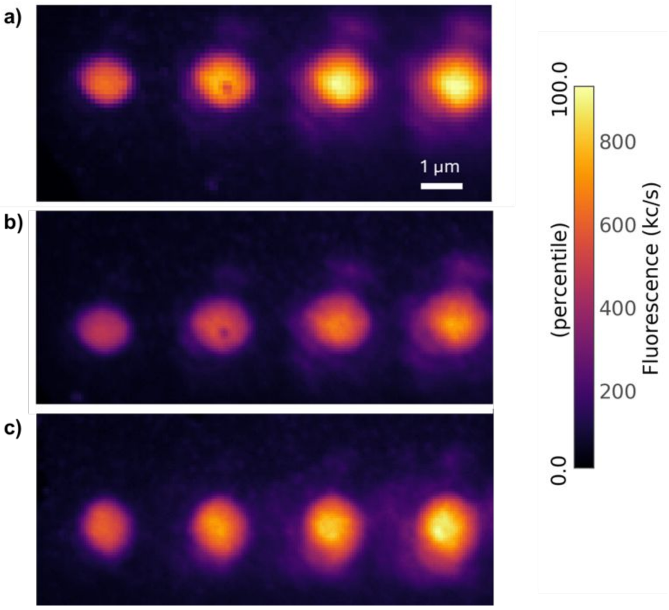

Fig. S3 a) PL map acquired in the area implanted with MgH^- ions and processed with a 405 nm laser (11 mW optical power) without any intermediate surface treatment. The four bright spots correspond to different laser processing exposure times (from left to right): 1, 5, 15, 30 min. **b)** PL map of the same region following surface oxidation treatment. **c)** PL map of an array of photoactivated spots (same laser processing parameters) fabricated after the oxygen plasma treatment of the sample.

S4 - GR1 center photoactivation

It is worth mentioning that the same photoactivation behavior observed for the MgV^- and Sn-related emission was noticed in Mg-implanted diamond in the case of the GR1 center, i.e. a broad emission band with ZPL at 741 nm associated with the single vacancy in its neutral charge state V^0 (**Fig. S4**). For each of the activated spots the presence of the GR1 was assessed by acquiring PL maps at >700 nm emission wavelengths (**Fig. S4a**) and analyzing the corresponding spectral emission (**Fig. S4b**). While a direct interplay between the MgV and vacancy complexes cannot be substantiated on the basis of the available data, the GR1 spectral signature is not detectable right after the ion implantation and it can be identified only after laser processing, similarly to the above-considered cases. This finding is in line with the results obtained for the MgV^- photoactivation, suggesting that the vacancies are formed in their negative charge state (ND1 center) upon ion implantation. Notably, no GR1 features were observed neither before nor after laser processing in the Sn-implanted sample. Considering that both Samples A and B underwent the same post-implantation processing, the absence of the GR1 emission in this latter case could be tentatively ascribed to the difference in mass of the implanted ion species, i.e. MgH^- and Sn^-. The significantly larger mass of Sn^- ions could lead to the formation of extended complexes[23] at the expense of the optically-active single vacancy.

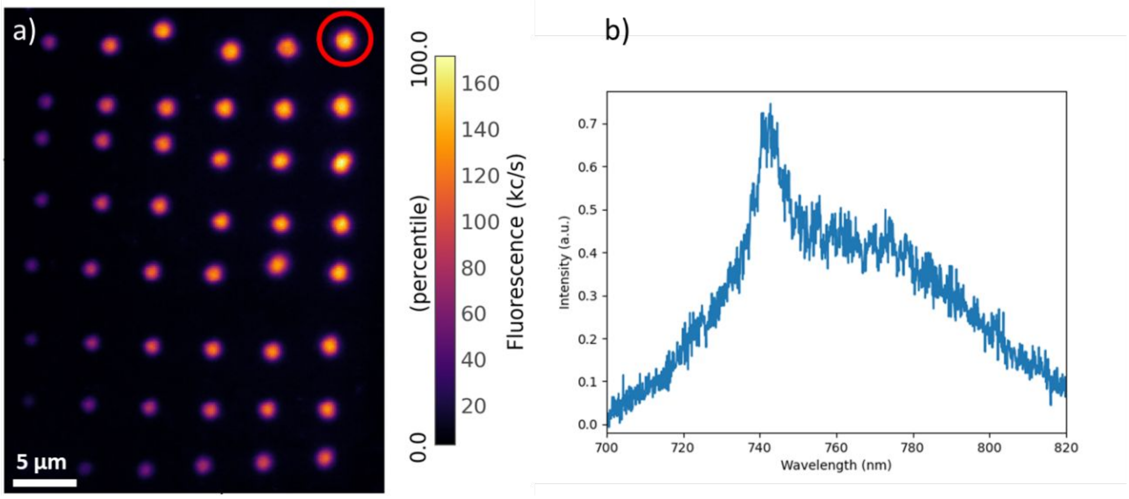

Fig. S4 a) PL confocal map acquired in the area implanted with MgH^- ions and processed with 445 nm laser. The map is acquired using a long pass filter with 700 nm cutoff wavelength. The photon collection thus only refers to the GR1 emission. **b)** Emission spectrum acquired from the spot circled in red in **Fig. S4a**.
